# Retraction: Anti Japanese encephalitis virus IgM positivity among patients with acute encephalitic syndrome admitted to different hospitals from all over Nepal

**DOI:** 10.1371/journal.pone.0187867

**Published:** 2017-11-06

**Authors:** The PLOS ONE Editors

After publication of this article, it came to light that the authors did not have the ethical approval required to conduct this study according to Nepalese research regulations. The ethics statement on the article states, "The research was approved by ethical committee of Nepal Public Health Laboratory, Kathmandu, Nepal." However, we were notified by officials at the Nepal Health Research Council (NHRC) that the Nepal Public Health Laboratory does not have an ethics committee and is not authorized to grant ethical approval for human subjects research in Nepal; such approval must come from the NHRC. While the authors contacted the NHRC about this study, they did not obtain ethical approval. Thus, this work did not meet Nepalese regulatory requirements or *PLOS ONE*'s publication criteria for human subjects research.

In light of the lack of ethical approval, the *PLOS ONE* Editors retract this article. We were unable to contact authors Anupama Bhattarai, Sailaja Adhikari and Mukunda Sharma about this retraction.
